# Trauma team training in Norwegian hospitals: an observational study

**DOI:** 10.1186/s12873-022-00683-9

**Published:** 2022-07-05

**Authors:** Ida Celine Bredin, Hedi Marina Joks Gaup, Guttorm Brattebø, Torben Wisborg

**Affiliations:** 1 Faculty of Health Sciences, Interprofessional Rural Research Team, University of Tromsø, The Arctic University of Norway, N-9600 Hammerfest, Norway; 2 Department of Anaesthesia & Intensive Care, Haukeland University Hospital, Bergen, Norway; 3 Department of Clinical Medicine, University of Bergen, Bergen, Norway; 4 Norwegian National Advisory Unit On Emergency Medical Communication (KoKom), Haukeland University Hospital, Bergen, Norway; 5 Department of Anaesthesia and Intensive Care, Hammerfest Hospital, Finnmark Health Trust, N-9613 Hammerfest, Norway; 6 Norwegian National Advisory Unit On Trauma, Division of Emergencies and Critical Care, Oslo University Hospital, N-0424 Oslo, Norway

**Keywords:** Trauma team, Trauma team training, Trauma system, Trauma plan, Norwegian hospitals, Norway

## Abstract

**Background:**

Traumatic injuries are a leading cause of deaths in Norway, especially among younger males. Trauma-related mortality can be reduced by structural measures, such as organization of a trauma system. Many hospitals in Norway treat few seriously injured patients, one of the reasons for development of the Norwegian trauma system. Since its implementation, there has been continuous improvement of this system, including trauma team training. Regular trauma team training is compulsory, with the aims of compensating for lack of experience and maintaining competence. The purpose of this study was to present an overview of current trauma team training activities in Norway.

**Methods:**

For this observational study, the authors developed an online questionnaire and mailed it to local trauma coordinators from 38 Norwegian hospitals—including four trauma centers and 34 acute hospitals with trauma function. The study was performed during April–June 2020, with a two-month response window. Trauma team training frequency was assessed in four predefined intervals: < 5, 5–9, 10–15 and > 15 times per year. The response rate was 33 of 38, 87%.

**Results:**

All responding hospitals conducted regular trauma team training. The frequency of training increased significantly from 2013 to 2020 (Chi square test, Chi^2^ 8.33, *p* = 0.04). All hospitals described a quite homogenous approach. The trauma centres trained more frequently as compared to the acute care hospitals (Chi square test, Chi^2^ 8.24, *p* = 0.04).

**Conclusions:**

All responding hospitals performed regular trauma team training using a homogenous approach, which is in line with previous assessments. We observed a minor improvement in frequency compared to prior assessments. Our findings suggest that Norwegian trauma teams likely maintain their competence through team training. All hospitals followed the current recommendations from the National Trauma Plan.

## Background

In Norway, approximately 300,000 people are injured and treated in hospitals every year [1]. Injuries are most commonly related to traffic (45%), followed by falls (42%) and sport and leisure (22%). In 2019, a total of 8788 trauma incidents were registered in the Norwegian National Trauma Register (NTR) [2]. Traumatic injuries take 2000 lives per year in Norway and are a leading cause of mortality among younger people. Notably, mortality related to accidents and injuries can be prevented. Structural measurements have proven effectiveness, such as the organization of trauma systems [1, 3].

Norwegian geographical and meteorological conditions are challenging, with long distances separating urban areas and sparsely inhabited rural settlements. This is further complicated by ever-changing weather conditions [4]. Previous studies have reported a higher risk of trauma-related mortality in rural areas compared to urban areas in Norway [5, 6]. Optimal trauma care in rural areas is challenging. Many hospitals treat a low annual number of seriously injured patients. According to figures from the NTR nearly half of hospitals (17 of 38) admitted less than 100 trauma patients in 2019 [2]. Moreover, among the total trauma admissions in these 17 hospitals, less than 10% of the admitted patients were seriously injured. Uleberg et al. [7] have described the challenges in Norway relating to the low number of trauma patients, and the uneven caseload between trauma centers and hospitals with acute trauma function. Multiple studies indicate that these factors should be considered in future modifications of the Norwegian trauma system [5, 7]. Geography, however, cannot be modified.

In 1997, the Better & Systematic Team Training (BEST) Foundation was established as a local initiative from two hospitals [8, 9]. The aim of this foundation was to make trauma team training a regular activity in Norwegian hospitals, with the initial focus on rural hospitals with low caseloads of severely injured trauma patients. This local initiative further influenced the improvement of trauma care throughout Norway. Ten years later, a multi-professional group prepared a report with the intention of implementing a Norwegian trauma system [10]. The aim of a trauma care system is to reduce trauma-associated mortality and morbidity. Several publications show that the implementation of trauma care systems has beneficial effects in terms of reducing these outcomes [3, 11].

Within the trauma system, Norwegian hospitals are divided into two levels: trauma centers and hospitals with acute care trauma function. The trauma system is based on the National Trauma Plan, which contains a set of predefined criteria for the involved hospitals, including some additional requirements for the trauma centers [10]. These requirements include regular trauma team training. Based on findings in the NTR report, the vast majority of trauma patients are first admitted to local hospitals with acute care trauma function, where there may be a lack of real-life experience with serious trauma patients [2]. Therefore, the National Trauma Plan requires regular trauma team training to maintain competence [10, 12]. This training is not detailed as to duration in hours but should contain a well-described simulation-based education in non-technical skills and crew resource management principles for all team members [8, 10].

Since its implementation, there have been continuous improvements of the trauma system and trauma team training in Norway [13–15]. In 2013, Dehli and colleagues assessed the current state of trauma system implementation [15]. Trauma team training was one of the 17 predefined trauma system criteria. Overall, the majority of the hospitals fulfilled this requirement. Additional data regarding trauma team training were simultaneously collected. The results showed that 37 (95%) of 39 responding hospitals performed regular trauma team training with varying frequency.

In the present study, we aimed to present an overview of trauma team training in Norway; to assess the regularity, frequency, and approach; and to review whether the hospitals follow the recommendations.

## Method

### Study setting

This study was conducted in Norway, which has an area of 384,484 square kilometers and 5,367,580 inhabitants as of 2020 [16, 17]. The Norwegian healthcare system comprises four regional health authorities. This study focused on the Norwegian trauma system, an essential part of the national healthcare system [10].

### Informants

Trauma coordinators from 38 Norwegian hospitals; four trauma centers and 34 acute hospitals with trauma function were invited to participate in the survey. This group of informants was chosen as they have the general overview regarding trauma team training in Norway.

### Study design and data collection

This observational study was based on an online questionnaire developed by the authors to gain an overview on trauma team training in Norway. Relevant questions were discussed with subject experts and based on a previous similar questionnaire from 2013 [15], with emphasis on simple questions based on the National Trauma Plan. As an example, the trauma team training frequency were given as four predefined intervals: under 5, 5–9, 10–15 and over 15 times per year. The questionnaire included questions about the participating trauma coordinators’ backgrounds, including the hospital they worked at and their involvement in trauma team training. Concerning trauma team training, we obtained information about frequency, method, experience, and allocated time. The questionnaire included both multiple-choice questions and open-ended questions and was pilot-tested by the trauma coordinator in Tromsø and the regional trauma coordinator of Northern Norway, to reduce the risk of systematic errors. In April 2020, potential participants were sent mail including information and an invitation to participate. During a 2-months’ collection window, the potential participants received three reminders. This study was approved by the Data Protection Officer in Finnmark Hospital Trust (RN: 2019/5022).

### Data analysis and statistical methods

The questionnaire had an integrated branching function, in which the data were categorized broadly based on former answers. This automatic categorization was followed by manual analysis. To reduce the risk of confirmation bias, two authors each independently went through the data material prior to joint review. Descriptive data are presented as proportions and absolute numbers. Trauma team training frequency was compared statistically by Chi square test with *p* = 0.05 as chosen level of significance. Each of the four possible reporting intervals was assigned the median value in the interval and the interval “ > 15” was assigned the value 25.

## Results

Of the 38 hospitals approached, five hospitals with acute trauma function did not respond, resulting in a response rate of 87%. All 33 responding trauma coordinators answered that they arranged or planned trauma team training alone or in collaboration with a colleague, and 28 (85%) of 33 facilitated the practical trauma team training.

**Fig. 1:**
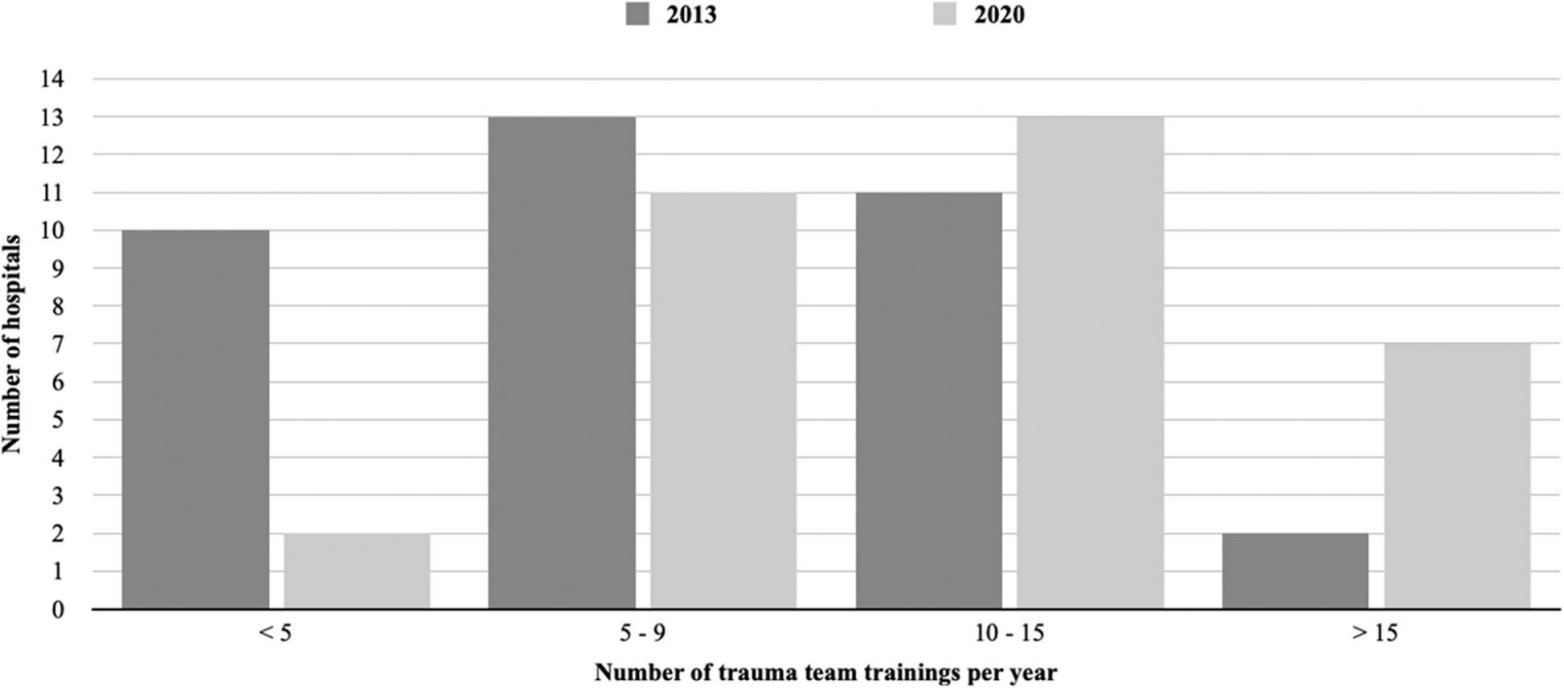
Distribution of trauma team training frequency per year in 2013 and 2020. Column height represents the number of hospitals per category

**Fig. 2:**
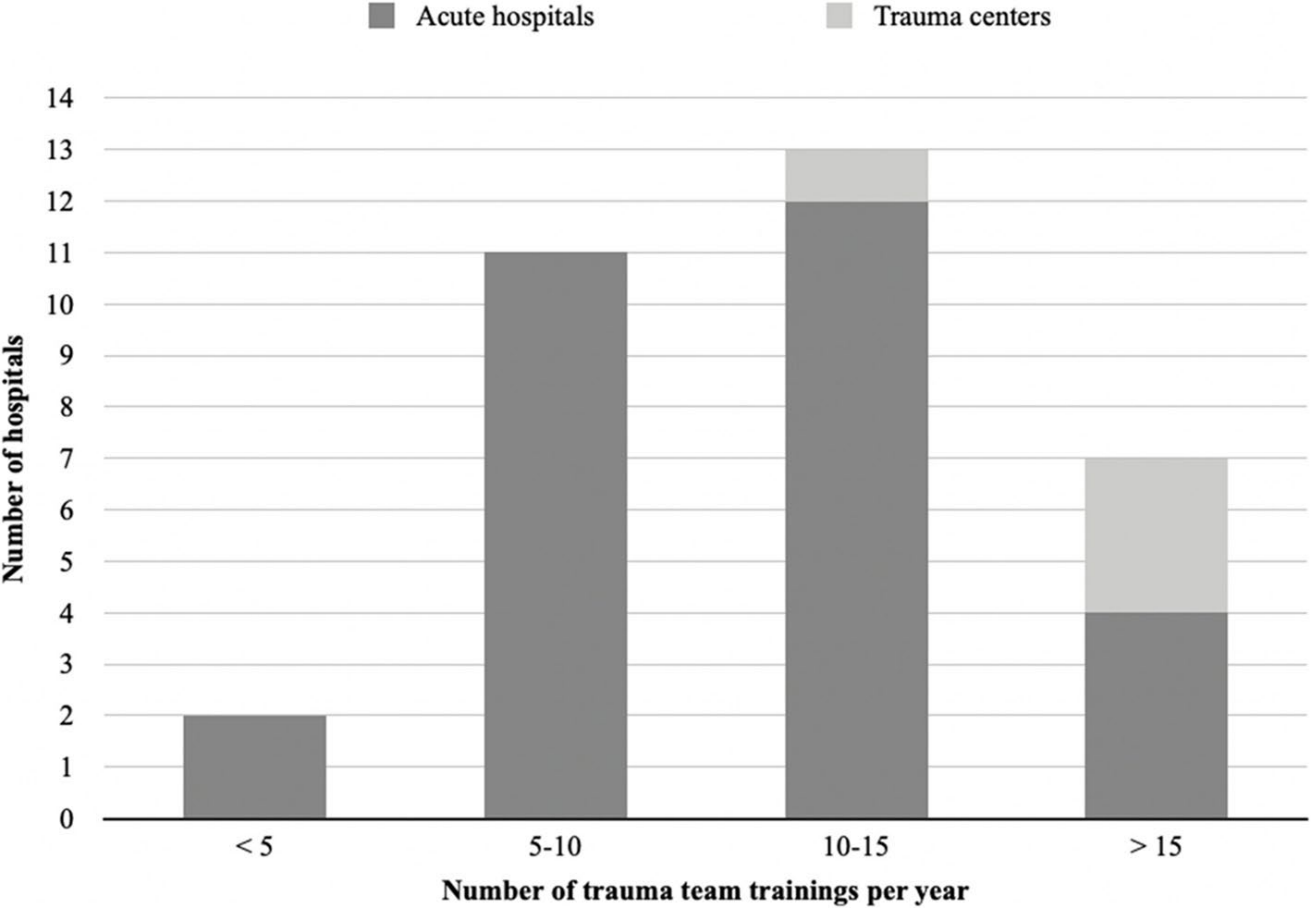
Annual trauma team training in Norwegian hospitals in 2020 in acute care hospitals (dark grey) and trauma centers (light grey)

All hospitals trained regularly. Figure 1 shows the difference between hospitals in the annual number of training sessions. The figure also displays historical data from 2013, provided by the authors of the study [15]. The frequency of training increased significantly from 2013 to 2020 (Chi square test, Chi^2^ 8.33, *p* = 0.04) On average, trauma team training sessions lasted for 30–60 min. Some hospitals reported that the trauma team training duration was affected by organizational factors, such as available time and staffing. The duration was also impacted by the choice of cases and the team’s needs. Of the 33 hospitals, 29 (88%) organized pre-training courses that were based on cases and focused on handling a patient in a trauma care setting. Figure 2 shows the annual trauma team training frequency in Norwegian hospitals in 2020 in acute care hospitals and trauma centers. The trauma centres trained more frequently as compared to the acute care hospitals (Chi square test, Chi^2^ 8.24, *p* = 0.04).

The majority of hospitals in this study had a common approach to their trauma team training. Many hospitals conducted training using educational material, including presentations, from the BEST foundation. The theme, cases, and priorities were usually based on the specific needs of the team and hospital. It was considered important to employ a realistic approach to trauma team training. Therefore, the hospitals chose real-life cases as background for simulation scenarios in the trauma room. Training was usually started with a trauma alarm, without any prior warning. One hospital sent advance information about the trauma team training, including learning objectives. The team responded as usual and gathered in the trauma room. There, they received information about the trauma team training, the case was presented, and a simulated patient brought to the room. Of the 33 hospitals, 11 (33%) always used a live human simulated patient; 19 (58%) used either a human simulated patient or a mannequin, depending on accessibility; and the remaining 3 (9%) always used a mannequin. At least one facilitator followed the team through the training. In most hospitals, the facilitator presented the patients’ vitals and clinical responses. Some hospitals used SimPad as a device for presenting vital signs. All hospitals concluded the training with a debriefing, to evaluate the team effort. Many of the hospitals conducted two consecutive rounds of training, with different cases or a higher difficulty. Of the 33 hospitals, 32 (97%) reported that the trauma team seemingly functioned better after regular trauma team training.

## Discussion

All hospitals in this study performed regular trauma team training using a homogenous approach, but with varying frequency.

In 2013, it was found that 37 (95%) of 39 Norwegian hospitals performed regular trainings, also with varying frequency [15]. Our findings confirm that there has not been any training fatigue and suggest that Norwegian hospitals still view trauma team training useful and as a priority. There have likely been minor changes and adaptations to adjust training to the clinical real-life needs and possibilities during recent years. The findings of the present study indicates that there have been improvements over the past five years. In 2006, Isaksen et al. concluded that Norway had efficiently implemented an improved trauma system compared to in 2000 [13]. At that time, half (54%) of the hospitals performed regular trauma team training, leaving ample room for improvement.

In 2000, one study claimed that it takes decades to identify significant effects on mortality after implementation of organized trauma systems [3]. This must be remembered when assessing the effects of more regular trauma team training in the Norwegian trauma system. Our results illustrate a prominent shift in the frequency of trauma team training over recent years, particularly in the groups that trained less than 5 or over 15 times per year. Several hospitals reported that they performed trauma team trainings more often in 2020 compared to in 2013. This higher frequency of training may have a greater impact on maintaining trauma care skills.

Jeppesen et al. report that as of 2020, few studies have examined trauma team training in the Nordic countries [18]. The majority of such studies have been observational studies that lack significant evidence. Our present study is an observational study that addressed the status of trauma team training in Norway in 2020. The last time this theme was well examined was in 2015 [15].

The participating hospitals reported rather homogenous methods for trauma team training, including realistic simulation sessions based on real-life cases. A systematic review of trauma team training found it difficult to compare the studies due to variations in training durations, combinations of methods, focus areas, and numbers of participants [19]. The authors concluded that simulation-based trauma team training results in significant improvement of the trauma team and discussed the need for more comprehensive randomized studies to evaluate the optimal approach for trauma team training [19]. Notably, multiple studies have shown positive effects and benefits from trauma team training [19–22].

It is difficult to define the best approach for trauma team training. The vast majority of studies have individually assessed the effects of various trauma team training programs. There is a lack of studies on determining the optimal approach, or that compare alternative approaches to trauma team training. The National Trauma Plan recommends that relevant personnel should participate in trauma team training at least once a year [10]. Most hospitals in our study arranged more than one trauma team training per year, but our data do not show whether each individual team member participated in multiple trainings per year. However, our results indicate that most hospitals arranged enough trauma team trainings per year for individuals possibly to participate in multiple sessions per year. The National Trauma Plan requires trauma team training at least once annually for all team members, for the purposes of compensating for a lack of real-life experience and maintaining a satisfactory level of competence [10]. This is checked by the regional trauma coordinators and is part of the annual quality control of acute hospitals with trauma function and trauma centers. Falcone et al. demonstrated that monthly simulation-based trauma team training over a year resulted in a significantly improved trauma team function [21]. Another study found that all of the observed improvements declined following cessation of the trauma team training simulation program [23]. Thus, on-going trauma team training is necessary to sustain the improvements. Some important factors that help to maintain regular trauma team training include local enthusiasts, administrative support, strategic planning, and facilitators [19, 24]. Future research should focus on determining the optimal duration, methods, and frequency of regular trauma team training [19, 20]. Ideally, trauma team training should be assessed by well-defined quality indicators like mortality, time to interventions, length-of-stay etc. This was not possible in the present study. It would be of value to evaluate the Norwegian trauma team training more in-depth in the future, including potential effects on mortality and correlations with volume and exposure.

### Limitations

The present respondents were limited to one local trauma team coordinator at each hospital, and the answers were not validated against other possible relevant sources. This methodology has previously been used for similar purposes in Norway. Our study had a somewhat lower response rate of 87%, but our findings are consistent with previous reports. Finally, the answers were not followed-up after data collection.

## Conclusion

All hospitals included in this study performed regular trauma team training. Since 2013, there has been a significant increase in the reported frequency of training. These findings are encouraging, especially for healthcare providers who treat a low caseload of trauma patients and hence lack real-life experience. The present results indicate that Norwegian trauma teams likely manage to maintain their competence, and that the requirements in the national trauma plan, in combination with meticulous assessments by regional trauma coordinators, keeps training activities ongoing. We found no signs of training fatigue. Future research should focus on potential effects on mortality and correlations with volume and exposure to identify the optimal approach for training, in terms of frequency, duration, methodology, and content.

## Data Availability

The datasets generated and analysed during the current study are not publicly available due to confidentiality of individual hospitals, but are available from the corresponding author on reasonable request.
